# Pseudohypoparathyroidism during pregnancy and the postpartum period: A case series of five patients

**DOI:** 10.3389/fendo.2022.1050305

**Published:** 2022-11-16

**Authors:** Jia-Jia Wang, Yi Yang, Ya-Bing Wang, An Song, Yan Jiang, Mei Li, Wei-Bo Xia, Yan-Ping Liu, Ou Wang, Xiao-Ping Xing

**Affiliations:** ^1^ Department of Endocrinology, Key Laboratory of Endocrinology, National Health Commission, Peking Union Medical College Hospital, Peking Union Medical College, Chinese Academy of Medical Science, Beijing, China; ^2^ Department of Clinical Nutrition, Peking Union Medical College Hospital, Peking Union Medical College, Chinese Academy of Medical Science, Beijing, China

**Keywords:** pseudohypoparathyroidism, pregnancy, lactation, serum calcium, serum PTH, treatment

## Abstract

**Objectives:**

Pseudohypoparathyroidism (PHP) is a rare disease, especially when combined with pregnancy. We aimed to explore the changes in serum calcium/parathyroid hormone (PTH) level and medical treatment in a case series of PHP during pregnancy and the postpartum period.

**Methods:**

A total of five PHP patients with six pregnancies were enrolled. The classification of PHP was based on (epi)genetic analysis. Clinical characteristics, biochemical indices, and treatment strategies before, during, and after pregnancy were retrospectively collected.

**Results:**

All patients received calcium and vitamin D agents with nearly normal serum calcium before pregnancy except patient 2 who was found hypocalcemic during gestation. All patients chose Cesarean section, and one suffered preterm delivery due to oligoamnios. The neonatal birth weight ranged from 2,250 to 4,300 g, and all neonates were free of hypocalcemia-related symptoms. The change in calcium metabolism was inconsistent including stable, improved, or worsened during pregnancy. Serum PTH level remained low in the first two trimesters in patients with stable and improved conditions while increased in the last two trimesters in patients with a worsened condition. Serum calcium changed inconsistently while PTH increased consistently during lactation. For patients who did not breastfeed, calcium homeostasis improved after delivery.

**Conclusion:**

Calcium homeostasis and medicine dosage changed differently in PHP patients during pregnancy and lactation. However, most patients had good pregnancy outcomes. Serum PTH levels might predict changes in calcium metabolism during pregnancy.

## 1 Introduction

Pseudohypoparathyroidism (PHP) is a rare endocrine disease characterized by resistance to the action of the parathyroid hormone (PTH), which is divided into PHP1 caused by (epi)genetic defects of GNAS gene which encodes stimulatory guanine nucleotide-binding protein (Gsα). PHP1a and PHP1b are the main subtypes of PHP1. PHP1a is caused by heterozygous loss-of-function mutations in maternally inherited *GNAS* exons 1–13, while PHP1b is caused by abnormal methylation at differentially methylated regions (DMRs) of the *GNAS* gene. Aside from PTH resistance, PHP1a patients also develop resistance to other hormones, such as thyroid-stimulating hormone (TSH), calcitonin, and growth hormone-releasing hormone (GHRH). In addition, patients with PHP1a can display Albright hereditary osteodystrophy (AHO) features characterized by short stature, brachydactyly, obesity with a round face, and heterotopic ossifications. PHP1b patients mainly manifest PTH resistance and are used to be considered free of the AHO phenotype. Recent studies showed that some PHP1b patients also develop TSH resistance and some AHO features, indicating an overlap between clinical features of both subtypes of PHP1 (1).

Since PTH is one of the most essential peptide hormones for calcium and phosphorus homeostasis, patients with PHP suffer from hypocalcemia, hyperphosphatemia, and high serum PTH level. At present, the treatment for PHP includes calcium and active vitamin D to maintain a normal serum calcium level and reduce the PTH level to normal range as much as possible (1).

Pregnancy and lactation are two periods in which calcium homeostasis changes greatly. During pregnancy, intestinal calcium absorption increases to ensure adequate fetal skeleton mineralization, with approximately 80% of the mineral accruing in the fetus in the third trimester (2). Many calcium-regulated hormones change markedly during this period, including PTH, PTH-related peptide (PTHrP), and calcitriol. According to previous studies, serum PTH typically declines to low levels at the early stage and then return to the mid-normal range by term when the calcium intake is sufficient. This suppression may not occur (or even secondary hyperparathyroidism may develop) in women whose calcium intake is insufficient (2). The PTHrP level progressively increases to reach peak levels in the third trimester, which is most likely secreted by the placenta and breasts. The calcitriol level begins to increase in the first trimester and may reach triple or more of the preconception level by the third trimester. However, the ionized or albumin-corrected serum calcium level is stable. Different from the pregnancy period, which relies mainly on increased intestinal calcium absorption to meet fetal calcium needs, increased skeletal resorption contributes to the majority of calcium requirements during lactation. This is consistent with the decline of serum calcitriol level to normal concentration. The PTH level is still typically suppressed toward the lower end of the normal range during lactation, while some studies have reported increased PTH in women from regions of Africa and Asia in which low calcium and vitamin D intakes are more prevalent. The PTHrP level remains high and the ionized or albumin-adjusted calcium level is normal to slightly increased in breastfeeding women (2).

For patients with PHP, the change in calcium metabolism during gestation and lactation is different from that of unaffected women. It is important to adjust medicine dosage in time to maintain calcium homeostasis for both maternal and fetal health. However, according to the limited previous case reports, the change in serum calcium level in PHP patients during pregnancy is inconsistent and the requirements of medication can change dramatically (3). Some patients had improved outcomes during pregnancy with abated hypocalcemic symptoms, normocalcemia, decreased to near-normal PTH level, and discontinuation of supplemental vitamin D agents (4), while other reports showed that the dose of calcium and/or active vitamin D had to be increased in PHP women during pregnancy (5–7). The data regarding mineral homeostasis in PHP patients during lactation are extremely rare. Only one case report published in 2017 was found (8).

Due to the rarity of the disease, the mechanism of the different reactions of PHP patients to pregnancy is still unknown. The aim of this study was to report a case series of pregnant women affected by PHP to help evaluate the clinical and biochemical course and pharmacological management during pregnancy and the postpartum period.

## 2 Subjects and methods

### 2.1 Subjects

PHP patients who were followed at the Endocrinology Clinic of the Peking Union Medical College Hospital (PUMCH) and pregnant during 2010 and 2021 were included in this study. The diagnostic criteria of PHP were PTH resistance and/or ectopic ossifications, and/or multiple hormone resistance including TSH resistance, and/or AHO (1). The further classification relied on (epi)genetic analysis of the *GNAS* gene, which was conducted and published previously (9). A total of five patients, including six pregnancies, were finally enrolled in the study. Three of them were diagnosed as PHP1b, and one was PHP1a. Patient 5 did not undergo gene detection, so her PHP type was not confirmed.

### 2.2 Data collection

#### 2.2.1 Clinical investigation

All of the clinical information was retrospectively obtained from the Hospital Information System of PUMCH. The PHP-related information included onset age, onset symptoms (e.g., muscle cramps, paresthesia, and seizures), treatment (dose of vitamin D agents and calcium), and complications of PHP (e.g., cataract screened by a slit lamp, renal stones/calcification screened by abdominal ultrasound, and intracranial calcification evaluated by cranial computed tomography). Pregnancy- and postpartum-related data included age of pregnancy, duration of the disease, maternal comorbidities and complications during pregnancy, pregnancy outcomes, delivery mode, birth weight, neonatal symptoms, and duration of lactation.

#### 2.2.2 Laboratory examinations

Serum biochemical indices, including total calcium (Ca), phosphorus (P), alkaline phosphatase, creatinine, and alanine aminotransferase, were measured using a Beckman automatic biochemical analyzer (AU5800; Beckman Coulter, CA, USA). The serum PTH level and total 25-hydroxyvitamin D (25OHD) level were measured using an electrochemiluminescence immunoassay (e601; Roche Cobas, Germany). Calcium homeostasis can be divided into three conditions based on the changes in serum calcium and drug dosage, including improved, worsened, and stable. Given that the serum total calcium level can be slightly decreased due to the physiological reduction of the serum albumin level, patients whose serum calcium level declined obviously (defined as a decline of >0.20 mmol/L) with stable medicine dosage were described as worsened. Patients who had increased serum calcium level with stable/reduced medicine requirement were described as improved. Patients with stable serum calcium levels (including patients with a decline in the serum total calcium level <0.2 mmol/L) and unchanged drug dosage were described as stable (10).

#### 2.2.3 Molecular analysis

##### 2.2.3.1 DNA extraction

Genomic DNA was extracted from peripheral leukocytes using the QIAGEN DNA extraction kit (QIAGEN, Hilden, Germany) according to the manufacturer’s instructions.

##### 2.2.3.2 Detection of *GNAS* mutations

The primers for *GNAS* were previously described (11). Sequencing of *STX16 and GNAS* was performed using the chain-termination method on an automatic sequencer (Applied Biosystems 3730 Genetic Analyzer, Foster City, CA, USA). Mutation taster (http://mutationtaster.org/) was used to predict the pathogenicity of the mutations.

##### 2.2.3.3 Analysis of *GNAS*-A/B:TSS-DMR, *GNAS*-NESP : TSS-DMR, *GNAS*-AS1:TSS-DMR, *GNAS*-XL : Ex1-DMR, and *STX16*

Methylation-specific multiple ligation-dependent probe amplification (MS-MLPA; MRC-Holland, Amsterdam, Netherlands) was performed to ascertain the methylation status of *GNAS* DMRs. Sporadic PHP1b was defined as broad alterations in methylation involving two or more DMRs, while familial PHP1b was defined as isolated loss of methylation (LOM) at *GNAS*-A/B:TSS-DMR, which was mostly due to deletion in the *STX16* gene.

## 3 Results

### 3.1 Case histories of the five patients

#### 3.1.1 Patient 1

Patient 1 was found hypocalcemic with unknown PTH level when she was 28 years old. When she was 31 years old, she came to our clinic due to the unconscious twitching of her fingers. Physical examination showed her height of 155 cm, weight of 61 kg, round face, and short neck without brachydactyly. Both Chvostek sign and Trousseau sign were positive. Blood testing showed severe hypocalcemia and elevated PTH level (**Table 1**). After (epi)genetic analysis, she was diagnosed with sporadic PHP1b (**Table 1**) and treated with calcium and vitamin D agents.

**Table 1:** General characteristics of the five patients.

Variables	Pt 1	Pt 2	Pt 3	Pt 4	Pt 5-1	Pt 5-2
PHP type	1b	1a	1b	1b	/	/
(epi)genetic data^*^	GOM at *GNAS-NESP* LOM at *GNAS-XL*, *GNAS-AS1*,*GNAS-A/B*	*GNAS*: Exon1:c.1247G>A	GOM at *GNAS-NESP* LOM at *GNAS-XL*, *GNAS-AS1*,*GNAS-A/B*	GOM at *GNAS-NESP* LOM at *GNAS-XL*, *GNAS-AS1*,*GNAS-A/B*	/	/
**Onset age (y)**	28	9	9	3	12	12
**Onset symptom**	none	convulsion	convulsion	convulsion	muscle cramp	muscle cramp
**Biochemical indices at the first visit** ^$^						
Serum Ca (mmol/L)	1.45	1.62	1.65	1.93	1.80	1.80
Ionized Ca (mmol/L)	0.74	/	/	0.86	0.68	0.68
Serum P(mmol/L)	1.77	2.04	1.72	1.88	2.10	2.10
Serum PTH(pg/ml)	334.6	188.2	314	731	518	518
24hUCa(mmol)	0.44	/	1.28	/	1.55	1.55
**Complications**						
Cataract	–	/	–	+	/	/
Renal stones	–	/	–	–	–	–
Intracranial calcification	+	/	+	+	+	+
**Subclinical hypothyroidism**	+	+	+	+	+	+
**Levothyroxine (μg/day)^#^**	50	82.14	107.14	67.5	50	0
**Pregnant age (y)**	32	29	24	27	22	27
**Pregnancy outcome**						
Full-term delivery^^^	N	Y	Y	Y	Y	Y
C-section	Y	Y	Y	Y	Y	Y
Birth weight (g/SD)	2250/ -0.75	3000/ 0.21	3600/ 1.09	3500/ 0.82	3600/ 0.64	4300/ 2.97
Neonatal serum Ca	normal	/	/	normal	normal	normal
**Lactation period (m)**	0	/	2	0	12	9

Pt, patient; PHP, pseudohypoparathyroidism; GOM, gain of methylation; LOM, loss of methylation; Ca, calcium; P, phosphorus; PTH, parathyroid hormone; UCa, urine calcium excretion; C-section, Cesarean section; N, no; Y, yes; y, year; m, month.

/, data unavailable; -, negative; +, positive.

^*^Patients 1–4 did not have heterozygous deletions at the *STX16* gene.

^$^Biochemical indices at the first visit time in our clinic.

^#^Levothyroxine dosage at the last visit time during the third trimester.

^^^Full-term delivery was defined as gestation period equal to or more than 37 weeks.

Normal reference ranges for indices: serum Ca, 2.13–2.70 mmol/L; ionized Ca, 1.08–1.28 mmol/L; serum P, 0.81–1.45 mmol/L; serum PTH, 13–65 pg/ml; 24hUCa, <7.5 mmol.

**Figure 1:**
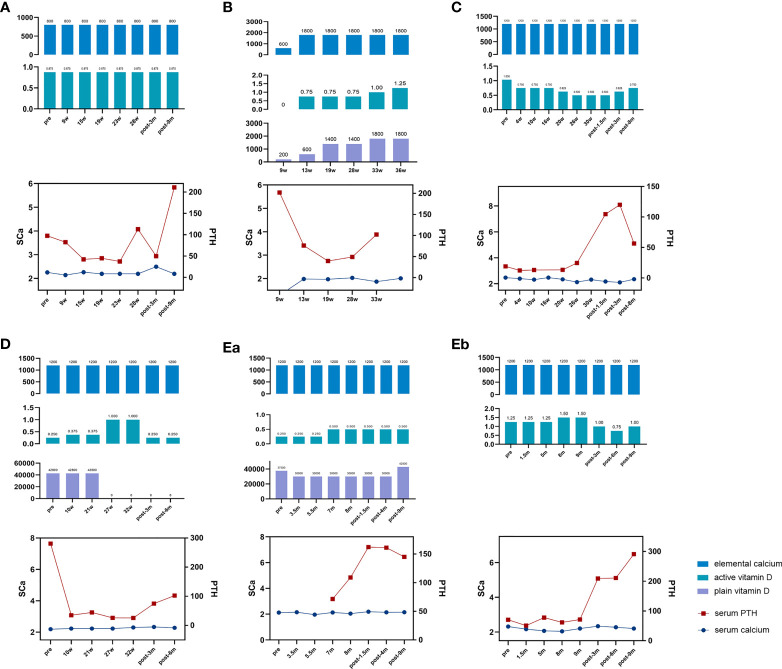
Changes in biochemical indices and medicine dosage of the patients. Elemental calcium (mg); active vitamin D, calcitriol (μg); plain vitamin D, calciferol/cholecalciferol (IU); serum PTH, serum parathyroid hormone level (ng/ml); SCa, serum calcium level (mmol/L); pre, prepregnancy; w, gestation week; post, postpregnancy; m, month after delivery. **(A)** Drug dosage, serum calcium, and PTH level of patient 1. **(B)** Drug dosage, serum calcium, and PTH level of patient 2. **(C)** Drug dosage, serum calcium, and PTH level of patient 3. **(D)** Drug dosage, serum calcium, and PTH level of patient 4. **(Ea)** Drug dosage, serum calcium, and PTH level of patient 5’s first pregnancy. **(Eb)** Drug dosage, serum calcium, and PTH level of patient 5’s third pregnancy.

She was pregnant at 32 years old. The detailed changes in serum calcium, 24-h urinary calcium excretion, and serum PTH level, as well as the adjustment of medication, were shown in **Tables 2** and **3** and **Figure 1A**, which revealed stable calcium metabolism. The serum 25OHD level was 46–62 ng/ml during her pregnancy period. Serum creatinine was within normal range all the time. A Cesarean section was carried out at week 35 due to gestational hypertension. The birth weight of the neonate was 2,250 g (-0.75 SD) with normal serum calcium level. She did not breastfeed after delivery (**Table 1**).

**Table 2:** Changes in serum calcium, 24-h urinary calcium, and serum PTH level during pregnancy and after delivery.

	Prepregnancy^*^			During pregnancy^#^									Postdelivery^$^(9 m)		
				First trimester			Second trimester			Third trimester					
Indices	SCa	24hUCa	PTH	SCa	24hUCa	PTH	SCa	24hUCa	PTH	SCa	24hUCa	PTH	SCa	24hUCa	PTH
**Pt 1**	2.25	2.49	97.5	2.14	/	82.7	2.21	3.79	59.4	/	4.71	/	2.34	4.10	130.4
**Pt 2**	/	/	/	1.28	/	202	1.98	10.66	57.9	1.97	7.09	75.7	/	/	/
**Pt 3**	2.47	3.95	19	2.36	4.50	12.75	2.31	2.90	18.9	2.32	4.41	/	2.21	2.35	93.8
**Pt 4**	2.19	3.71	281	2.23	5.67	34.9	2.23	4.35	35.3	2.30	6.91	25.6	2.31	8.51	88.8
**Pt 5-1**	2.12	2.32	/	/	/	/	2.06	2.07	/	2.09	2.37	90.3	2.16	1.82	156
**Pt 5-2**	2.32	5.47	70.4	2.17	3.48	50.8	2.07	6.90	77.8	2.12	4.00	66.5	2.27	4.37	236.8

Pt, patient; SCa, serum calcium (mmol/L); PTH, parathyroid hormone (pg/ml); 24hUCa, 24-h urinary calcium excretion (mmol); m, month.

/, data unavailable.

^*^Serum calcium, 24-h urinary calcium excretion, and serum PTH level at the last visit time before pregnancy.

^#^Average value of serum calcium, 24-h urinary calcium excretion, and serum PTH level during the three trimesters: the first trimester was before week 13; the second trimester was week 13 to week 27; the third trimester was week 28 to delivery.

^$^Average value of serum calcium, 24-h urinary calcium excretion, and serum PTH level within 9 months after delivery.

Normal reference ranges for indices: serum Ca, 2.13–2.70 mmol/L; serum PTH, 13–65 pg/ml; 24hUCa, <7.5 mmol.

**Table 3:** Changes in medicine dosage during pregnancy and after delivery.

	Prepregnancy^*^			During pregnancy^#^									Postdelivery^$^(9 months)		
				First trimester			Second trimester			Third trimester					
Indices	Ca(mg/day)	Active D(μg/day)	Plain D(IU/day)	Ca(mg/day)	Active D(μg/day)	Plain D(IU/day)	Ca(mg/day)	Active D(μg/day)	Plain D(IU/day)	Ca(mg/day)	Active D(μg/day)	Plain D(IU/day)	Ca(mg/day)	Active D(μg/day)	Plain D(IU/day)
**Pt 1**	800	0.875	0	800	0.875	0	800	0.875	0	/	/	/	800	0.875	0
**Pt 2**	/	/	/	600	0	200	1,800	0.75	1,000	1,800	1.00	1,667	/	/	/
**Pt 3**	1,200	1.036	0	1,200	0.75	0	1,200	0.625	0	1,200	0.5	0	1,200	0.625	0
**Pt 4**	1,200	0.25	4,2800	1,200	0.375	4,2800	1,200	0.688	2,1400	1,200	1	0	1,200	0.25	0
**Pt 5-1**	1,200	0.25	3,7500	/	/	/	1,200	0.25	3,0000	1,200	0.5	3,0000	1,200	0.5	3,4300
**Pt 5-2**	1,200	1.25	0	1,200	1.25	0	1,200	1.25	0	1,200	1.5	0	1,200	0.92	0

Pt, patient; Ca, elemental calcium dosage; Active D, calcitriol dosage; Plain D, calciferol/cholecalciferol dosage.

^*^Drug dosage at the last visit time before pregnancy.

^#^Average drug dosage during the three trimesters: the first trimester was before week 13; the second trimester was week 13 to week 27; the third trimester was week 28 to delivery.

^$^Average drug dosage within 9 months after delivery.

Before this pregnancy, the patient experienced her first pregnancy at 28 years old and was found hypocalcemic during the pregnancy period. However, detailed information about that pregnancy was missing. Cesarean section was carried out, and a healthy male infant was delivered with a birth weight of 2,900 g.

#### 3.1.2 Patient 2

Patient 2 suffered from generalized convulsions from 9 years old and was treated with calcium supplements all the time. She first came to our clinic at 29 years old while at week 9 of her first pregnancy. Physical examination showed AHO phenotype, including a round face, short neck, and short metacarpals/metatarsals. Blood testing showed severe hypocalcemia with a high PTH level (**Table 1**) as well as vitamin D deficiency (serum 25OHD level was 12.9 ng/ml). Genetic testing supported a diagnosis of PHP1a (**Table 1**). As shown in **Tables 2** and **3** and **Figure 1B**, her calcium metabolism worsened during pregnancy with decreased serum calcium and increased medicine dosage. The serum 25OHD level rose to 30.1 ng/ml at week 33 after vitamin D supplementation. She did not come to our clinic after week 36, so the clinical data after that time were unavailable. She underwent Cesarean section at approximately week 37 due to fetal intrauterine hypoxia. She underwent surgery at the local hospital, and the neonatal Apgar score was not available. The neonatal birth weight was about 3,000 g (0.21 SD). Unfortunately, the baby died within several months due to an intestinal fistula.

#### 3.1.3 Patient 3

Patient 3 suffered convulsion and unconsciousness at the age of 9 years. In the beginning, she was diagnosed with hypoparathyroidism (HP) and treated with calcium and calcitriol. When she was 21 years old, she came to our clinic and was diagnosed with sporadic PHP1b on the basis of hypocalcemia, high PTH levels, and (epi)genetic testing (**Table 1**). She was pregnant at 24 years old. The serum 25OHD level was detected at week 4, and the result was 30.8 ng/ml. As shown in **Tables 2** and **3** and **Figure 1C**, her calcium homeostasis improved during pregnancy with a decreased medicine dosage and a stable serum calcium. A Cesarean section was carried out at week 39 due to oligoamnios, and the neonatal birth weight was 3,600 g (1.09 SD). The neonate was free of hypocalcemia-related symptoms, while the serum calcium level was not detected. She breastfed for about 2 months due to little milk quantity. After delivery, the drug dosage needed to be increased.

#### 3.1.4 Patient 4

Patient 4 suffered from intermittent convulsion from 3 years old and had seizure episodes with loss of consciousness and urinary incontinence from 10 years old. She came to our clinic at 20 years old due to intermittent convulsion. Sporadic PHP1b was diagnosed based on biochemical testing and (epi)genetic analysis (**Table 1**). She was pregnant at 27 years old. However, calcium metabolism was difficult to evaluate during pregnancy due to the change in vitamin D agents (**Table 3**; **Figure 1D**). Her serum 25OHD level was tested at week 4, and the value was 195 ng/ml. She delivered at week 39 by Cesarean section due to the cord around the neck. The neonatal birth weight was 3,500 g (0.82 SD), and the serum calcium was normal. The patient did not breastfeed due to little milk quantity, and her treatment needs decreased after delivery.

#### 3.1.5 Patient 5

Patient 5 suffered from muscle cramps at 12 years old. Blood testing showed hypocalcemia, high PTH level, and normal kidney/liver function (**Table 1**). However, the type of PHP was unclear due to the lack of (epi)genetic analysis. A family history of parathyroid disease was denied. Her first pregnancy was at 22 years old. During this pregnancy period, calcium metabolism worsened with obviously declined serum calcium and increased drug dosage (**Tables 2**, **3**; **Figure 1Ea**). The serum 25OHD level was not tested during this pregnancy period. A female neonate was delivered by Cesarean section at about week 41 due to decreased range of motion of the hips. The infant weighed 3,600 g (0.64 SD) with a normal serum calcium level. She breastfed for about 1 year with a small amount of milk. During lactation, the treatment needs increased because the PTH level obviously elevated.

She was pregnant again at 26 years old but suffered arrested fetal development in month 3 with an unknown reason. When she was 27 years old, she was pregnant for the third time. Serum 25OHD level was detected before this pregnancy, and it was 60.4 ng/ml. It was tested again at the eighth month during pregnancy, and the result was 46.5 ng/ml. Calcium metabolism also worsened during this pregnancy period (**Tables 2**, **3**; **Figure 1Eb**). She delivered at week 39 by Cesarean section. The infant weighed 4,300 g (2.97 SD), and the serum calcium level was at the lower part of the normal range. She breastfed for more than 1 year, and the amount of milk was more than that of her first lactation. The medicine dosage was reduced, and PTH level increased compared to that during her pregnancy.

### 3.2 General characteristics of the five patients

As shown in **Table 1**, a total of five patients (Pts) with six pregnancies were included in this study. The average onset age for the five patients was 12.2 years, and the mean disease course from onset to pregnancy was 14.7 years. Four of them had intracranial calcification except for Pt 2 with missing data. Cataract was found in one patient. In addition, all patients were found suffering from subclinical hypothyroidism (TSH resistance) before or during pregnancy and were suggested to have levothyroxine treatment (Pt 5 did not follow medical advice during her second pregnancy period).

### 3.3 Changes in biochemical indices and treatment needs during pregnancy and after delivery

As shown in **Figure 1**, during pregnancy, calcium homeostasis was described as stable in Pt 1 due to stable serum calcium level and drug dosage (**Figure 1A**), improved in Pt 3 due to increased serum calcium and reduced drug dosage (**Figure 1C**), and worsened in Pt 5 because of the obviously decreased serum calcium level and increased drug dosage during the two pregnancies (**Figure 1E**). For patients 2 and 4, the changes in PHP condition were difficult to evaluate due to the lack of prepregnancy data and the change in vitamin D agent type (**Figures 1B, D**).

All patients had normal 24-h urinary calcium level before pregnancy except patient 2 with missing data. Also, the 24-h urinary calcium level slightly increased during pregnancy compared to prepregnancy among all patients. Except patient 2, who did not accept standardized and regular treatments until week 9 of gestation, all patients had normal 24-h urinary calcium level during pregnancy and patient 4 suffered from hypercalciuria after delivery. Renal function remained normal in all patients.

The serum PTH level was obviously decreased in the first trimester. For patient 1, whose condition was stable during the whole gestation period, the PTH level further decreased in the second trimester. For patient 3, whose condition improved during pregnancy, the PTH level remained very low in the second trimester. And for the third pregnancy of patient 5, whose condition has worsened, the PTH level was slightly increased in the last two trimesters compared to that of the first trimester (**Table 2**; **Figure 1**).

Only two patients breastfed after three deliveries. Pt 3 breastfed for 2 months. At 1.5 months after delivery, a slight decrease in serum calcium level (from 2.32 to 2.17 mmol/L) and a marked increase in PTH level (from 24.5 to 104.7 pg/ml) were observed. Pt 5 breastfed for 12 months after her first pregnancy and more than 1 year after her third pregnancy. Increased serum calcium and PTH levels were found during these two lactation periods compared to those during pregnancy (**Table 2**; **Figure 1E**).

For patients who did not breastfeed (Pts 1 and 4), it seemed that the calcium condition changed consistently. The serum calcium level increased from 2.19 (week 28) to 2.49 mmol/L (3 months postpartum) with stable medicine and declined PTH level (from 113 to 49.9 pg/ml) in patient 1 (**Figure 1A**). Calcitriol was reduced from 1 μg/day (week 32) to 0.25 μg/day (3 months postpartum) with stable serum calcium and increased PTH level (from 25.6 to 75.1 pg/ml) in Pt 4 (**Figure 1D**).

### 3.4 Pregnancy outcomes

All patients chose Cesarean section due to variable reasons, and one suffered preterm delivery due to oligoamnios (Pt 1). The neonatal birth weight ranged from 2,250 to 4,300 g, and all neonates were free of hypocalcemia-related symptoms. Serum calcium levels were detected in four neonates, and all were in the normal range. Thyroid hormone was tested in all neonates using heel blood at birth. In addition, their thyroid functions were all in the normal range.

## 4 Discussion

PHP is a rare disease with a reported prevalence of about 1.2/100,000 (12). It can be challenging to manage especially during pregnancy. Information about calcium metabolism change and medicine dosage adjustment in such condition is scarce. Previously, only 12 cases with 14 pregnancies were reported. Our study reported five PHP patients with six pregnancies to show the changes in serum calcium/PTH level and medicine dosage during pregnancy and the postpartum period, as well as their pregnancy outcome, providing more information on this rare but important clinical entity.

The results showed that PHP patients can have different conditions in calcium homeostasis and treatment needs during pregnancy: improved, stable, or worsened, which was similar with previous case reports. According to previous studies, patients with improved outcome had some common characteristics: firstly, their calcium supplementation was high (usually more than 1 g/day); secondly, the PTH level was continuously low during the whole pregnancy period; thirdly, the serum calcitriol level increased 2–4-fold compared to prepregnancy during the entire gestation period (4). On the contrary, as for patients with worsened outcome, calcium supplementation was very low (<0.5 g/day) (5–7). In addition, the fall in albumin-adjusted calcium level in the latter half of the pregnancy period was accompanied by a declined calcitriol level and a risen PTH level (6). In normal women, the physiology of pregnancy means that the recommended dietary intake of calcium (1.25 g/day), combined with doubling of efficiency of intestinal calcium absorption, should be more than sufficient to meet the combined needs of the mother and fetus during the third trimester (13). So, as mentioned before, when the calcium intake is insufficient, pregnant women can develop secondary hyperparathyroidism to provide additional minerals. Since PHP patients are PTH resistant, it is not surprising that the PHP-related condition will worsen if calcium intake is inadequate during pregnancy. Another important factor that may affect the PHP condition during pregnancy is the calcitriol level, since the worsened patients had declined calcitriol levels. However, factors that influence the calcitriol level during pregnancy remain to be elucidated. According to this study, patient 5 had worsened outcome with elemental calcium supplementation of 1,200 mg/day. Thus, her worsened status during her two pregnancies might be due to the reduced serum calcitriol level. Regretfully, this index was not detected during her pregnancy. In terms of PTH level, our study showed that the decreased serum calcium level was accompanied by an elevated PTH level, which was consistent with previous studies. The PTH response to pregnancy in PHP patients was similar to that of a normal person, characterized by declining in the first trimester then possibly elevating in the third trimester due to further increased calcium demands. The low calcium intake, declined calcitriol level, and elevated PTH level might indicate the need to increase medicine dosage for PHP pregnant women.

As to the urinary calcium excretion, firstly, our results showed that the 24-h urinary calcium level was slightly increased during pregnancy compared to prepregnancy, which was similar to unaffected women and might be due to the increased intestinal calcium absorption. Secondly, different from patients with HP, who easily developed hypercalciuria under active vitamin D and calcium treatment, the 24-h urinary calcium excretion was within normal range in most patients with PHP. This indicated that the renal resistance to PTH in patients with PHP1 was limited to the proximal tubular cells and did not involve the distal tubular cells.

In the aspect of pregnancy and neonate outcomes, our study showed that all patients underwent Cesarean section due to variable reasons and most of them reached full-term delivery. In addition, all available serum calcium levels of neonates were within normal range. However, one neonate (the second child of patient 5) in the present study had macrosomia. Up to now, all of the PHP pregnant women reported in previous literature chose Cesarean section at full term while delivering neonates with normal birth weight (2,593–3,460 g) and mostly normal serum calcium level. The reasons for Cesarean section were the reduced pelvic size and decreased range of motion of the hips due to local ossifications. However, high birth weight had been reported in some previous cases with PHP1b caused by paternal uniparental disomy of chromosome 20 (14) and *STX16* microdeletion (15), which means that both sporadic and familial PHP1b patients could manifest as macrosomia themselves. In 2015, Bréhin et al. (16) reported 114 PHP1b patients (61 familial and 53 sporadic) and 12 PHP1a patients with their own birth conditions. Their results showed that PHP1a patients were free of intrauterine overgrowth, while PHP1b patients themselves had heavier birth weight than the normal population (Z-score of +0.73 ± 1.2, P < 0.0001). Moreover, familial PHP1b patients tended to have slightly heavier birth weight than sporadic PHP1b patients. In addition, familial PHP1b patients had heavier birth weight than their healthy siblings. These indicated that biallelic A/B expression might contribute to enhanced intrauterine growth through an unknown mechanism. Regretfully, Pt 5 and her second child did not undergo (epi)genetic analysis, so their PHP types were unknown. However, since she had PHP and her second child had macrosomia while the first child had normal birth weight, it was suspected that she and her second child had familial PHP1b. Thus, an (epi)genetic analysis for them is helpful, and the serum calcium and PTH levels of the second child should be monitored.

In the present study, two patients breastfed after delivery with different responses to breastfeeding. Patient 3 showed worsened response with a slight decrease in serum calcium level and marked increase in PTH level. Patient 5 showed an improved condition characterized by increased serum calcium level compared to the pregnancy period during both lactations. We had retrieved only one case report describing calcium homeostasis in PHP patients during the lactation period, which showed a stable serum calcium level and medicine dosage in a PHP1a patient with calcitriol 0.5 μg/day and calcium carbonate 2,500 mg/day (1,000 mg elemental calcium). The PTH level was not described (8). Since the elemental calcium intake was similar among the two patients in the present study and the patient in a previous case report, there should be other factors that influence calcium metabolism. Calcium homeostasis in lactation relies mainly on bone resorption mediated by PTHrP, which comes mainly from the mammary gland. In the present study, patient 3 had worsened calcium homeostasis during lactation with little milk amount and short breastfeeding duration. Whereas patient 5 had improved calcium homeostasis with more milk amount and a longer lactation duration than those in patient 3. So, the different change in calcium homeostasis between patient 3 and patient 5 in their lactation period might be related to the different amount of milk and PTHrP level. Hence, there might be some relationship between milk amount and PTHrP level. However, there was no convincing method to evaluate that the amount of milk and PTHrP level were not detected in both of these two studies. So, this assumption needs further exploration. Different from serum calcium level, the change in PTH level was consistent in the two patients’ three lactation periods. This phenomenon was also found in normal women who suffered from insufficient calcium intake when breastfeeding. So, it may be an indicator of the insufficient calcium intake.

The calcium metabolism for those PHP patients who did not breastfeed (patient 1 and patient 4) seemed to improve with elevated serum calcium level (patient 1) or declined medicine dosage (patient 4). This was inconsistent with a previous study that showed reduced serum calcium level and increased drug dosage after delivery (4). The underlying mechanism of the different response was unknown and needed further exploration. Genetic or ethnic differences may have some effect.

Our center has reported a case series about the change in calcium metabolism of HP patients during pregnancy and the lactation period (10). The results showed that HP patients could suffer from marked serum calcium fluctuations during these two periods and the risk of adverse pregnant outcomes was higher than that of a normal person. During pregnancy, hypercalcemia can occur and HP patients who had improved calcium metabolism might need to discontinue active vitamin D. While for HP patients who had worsened calcium homeostasis during pregnancy, a marked decrease in serum calcium level can be found and might lead to abortion/stillbirth. During lactation, most patients had improved calcium metabolism with increased serum calcium level. Some patients even developed hypercalcemia. There were some similarities and differences between PHP and HP during pregnancy and lactation periods. During the pregnancy period, on the one hand, both PHP and HP patients could experience opposite changes in calcium homeostasis as objectively improved or objectively worsened status. On the other hand, the serum calcium fluctuation and pregnant outcomes were different. Compared to HP, PHP patients seemed to have a much milder serum calcium fluctuation and better pregnancy outcomes. During the lactation period, the fluctuation of serum calcium level also seemed milder in PHP patients than that in HP patients. These indicated that PTH may still have some effect on calcium metabolism in PHP pregnant patients.

This study had some limitations. First, because of the retrospective nature, some data were missing and the follow-up time points could not be controlled. Second, neither the ionized calcium nor serum albumin level was routinely detected. Since the serum albumin level was reduced during pregnancy due to dilution, the total serum calcium level without albumin adjustment might underestimate true calcium conditions and influence the drug dosage adjustment. Third, some indices were not detected in the study, such as the maternal 25OHD, 1,25(OH)_2_D, and PTHrP levels, which restricted further exploration. Fourth, due to the rarity of PHP, especially combined with pregnancy, the sample size of the study was small, which cannot fully represent the changes in calcium metabolism in PHP patients during pregnancy and lactation. However, this study was still a relatively large single-center study that focused on PHP conditions among pregnant women, providing useful information on these clinical entities.

## 5 Conclusion

In conclusion, the present study described the changes in serum calcium level, PTH level, and treatment needs in PHP patients during pregnancy and after delivery. It confirmed that the calcium condition and drug dosage changed differently in PHP patients during pregnancy and lactation but were more stable than those in HP patients. Most patients had good outcome with full-term delivery and normal birth weight. During pregnancy, an elevated PTH level might predict a worsened calcium metabolism and increase of medicine requirement. It reminds clinicians to focus on PTH level and adjust medicine dosage in time to maintain normal calcium metabolism in PHP pregnant patients.

## Data availability statement

The original contributions presented in the study are included in the article/supplementary material. Further inquiries can be directed to the corresponding authors.

## Ethics statement

The studies involving human participants were reviewed and approved by The Institutional Review Board of Peking Union Medical College Hospital. The patients/participants provided their written informed consent to participate in this study. Written informed consent was obtained from the individual(s) for the publication of any potentially identifiable images or data included in this article.

## Author contributions

Study design: J-JW, OW, and X-PX. (Epi)genetic analysis: YY and Y-BW. Data collection: AS, YJ, ML, Y-PL, and W-BX. Data analysis: J-JW and OW. Drafting manuscript: J-JW and OW. Revising manuscript content: OW. Approving final version of manuscript: J-JW, YY, Y-BW, AS, YJ, ML, W-BX, Y-PL, OW, and X-PX. All authors contributed to the article and approved the submitted version.

## Funding

This study was supported by grants from the National Natural Science Foundation of China (No. 81873641 and No. 82070817) and the Chinese Academy of Medical Sciences (CAMS) Innovation Fund for Medical Sciences (CIFMS) (2017-I2M-1-001).

## Conflict of interest

The authors declare that the research was conducted in the absence of any commercial or financial relationships that could be construed as a potential conflict of interest.

## Publisher’s note

All claims expressed in this article are solely those of the authors and do not necessarily represent those of their affiliated organizations, or those of the publisher, the editors and the reviewers. Any product that may be evaluated in this article, or claim that may be made by its manufacturer, is not guaranteed or endorsed by the publisher.
